# SECTM1 promotes the development of glioblastoma and mesenchymal transition by regulating the TGFβ1/Smad signaling pathway

**DOI:** 10.7150/ijbs.84591

**Published:** 2024-01-01

**Authors:** Zhipeng Yao, Fan Zhang, Chenxue Qi, Cheng Wang, Min Mao, Chenhui Zhao, Min Qi, Zhichun Wang, Guoren Zhou, Xiaochun Jiang, Hongping Xia

**Affiliations:** 1 School of Chemistry and Chemical Engineering & Zhongda Hospital, School of Medicine, Advanced Institute for Life and Health, Southeast University, Nanjing 210096, China.; 2 The Translational Research Institute for Neurological Disorders, Department of Neurosurgery, the First Affiliated Hospital (Yijishan Hospital), Wannan Medical College, Wuhu 241000, China.; 3 Department of Gynecologic Oncology, Cancer Hospital of Shantou University Medical College, Shantou 515041, China.; 4 Department of Pathology, Nanjing Drum Tower Hospital Clinical College & Key Laboratory of Antibody Technique of National Health Commission & Jiangsu Antibody Drug Engineering Research Center, Nanjing Medical University, Nanjing, China.; 5 Institute of Pathology and Southwest Cancer Center, Southwest Hospital, Third Military Medical University (Army Medical University) and Key Laboratory of Tumor Immunopathology, Ministry of Education of China, Chongqing, 400038, China.; 6 Department of Oncology, Jiangsu Cancer Hospital, The Affiliated Cancer Hospital of Nanjing Medical University, Jiangsu Institute of Cancer Research, Nanjing 210009, China.

**Keywords:** SECTM1, glioblastoma, invasion, EMT

## Abstract

**Objective:** Secreted and transmembrane protein 1 (SECTM1) is a gene encoding a transmembrane protein. The role of SECTM1 in glioblastoma (GBM) is unclear. Here, we reported the abnormal expression of SECTM1 in GBM for the first time and studied the role and mechanism of SECTM1 in GBM.

**Methods:** qRT-PCR, Western blotting and immunofluorescence were used to detect the expression of SECTM1 in gliomas of different grades and GBM cell lines. After the knockdown of SECTM1 expression in cell lines by shRNA, the effect of SECTM1 in GBM cell lines was verified by CCK-8, Transwell, EdU and wound healing experiments. We further investigated the effect and mechanism of SECTM1 on GBM *in vitro* and *in vivo*. The effect of SECTM1 on glioma growth was detected by subcutaneous tumor xenografts in nude mice *in vivo*.

**Results:** The results showed that the knockdown of SECTM1 expression in cell lines significantly inhibited the proliferation, migration and invasion of GBM cells while inhibiting the progression of subcutaneous xenograft tumors in nude mice. However, the role and molecular mechanism of SECTM1 in GBM remain unclear. SECTM1 was found to promote GBM epithelial-mesenchymal transition (EMT) like processes. Bioinformatics analysis and Western blotting showed that SECTM1 regulates glioblastoma invasion and EMT-like processes mainly through the TGFβ1/Smad signaling pathway.

**Conclusion:** The low expression of SECTM1 has an inhibitory effect on GBM and is a potential target for GBM treatment. SECTM1 may also be a promising biomarker for the diagnosis and prognosis of GBM.

## Introduction

Glioblastoma multiforme (GBM) is a common malignant tumor in the central nervous system (CNS) and one of the diseases that seriously threaten human health^1^, ^2^. Because glioma cells are characterized by rapid proliferation, migration, and invasion, as well as the ability to stimulate angiogenesis, gliomas grow in an invasive and expansive manner and subsequently progress to higher grades^3^. The current standard treatment for patients with glioma is surgical resection, followed by radiotherapy and adjuvant chemotherapy^4^. As the highest glioma grade, GBM accounts for more than half of all gliomas and is essentially incurable^5^, ^6^. Due to treatment resistance and tumor recurrence, efforts have been made to identify the molecules that regulate the occurrence and development of GBM. These molecules play an essential role in regulating GBM migration and invasion and may serve as critical therapeutic targets^7^-^9^. Therefore, it is necessary to identify the therapeutic target molecules that regulate the development of GBM, provide additional therapeutic means for glioma patients, and develop effective targeted molecular therapy.

Secreted and transmembrane protein 1(SECTM1) is approximately 14 kb in length, encodes an 8 kb mRNA, and is translated as a type I transmembrane protein of size 27 kDa, present in both transmembrane and soluble forms, and its soluble form is a ligand for CD7^10^, ^11^. SECTM1 is expressed in various normal cells, including neutrophils, dendritic cells, and epithelial cells, but not in peripheral lymphocytes^12^. SECTM1 is highly expressed in many tumors, including melanoma^13^, and it is also highly expressed in breast and prostate cancers and some myeloid leukemias^10^, ^14^. The potential role of SECTM1, which is highly expressed in leukemia, is to bind to CD7 to activate T and NK cells and stimulate their proliferation^14^. The expression of SECTM1 in melanoma cells suggests that SECTM1 may play a role in regulating the tumor microenvironment by inducing monocyte function^13^. However, the biological significance and role of SECTM1 expression in GBM remain unclear.

In this study, we first demonstrated the role of SECTM1 in GBM and investigated its mechanism. We detected the expression of SECTM1 in glioma specimens of different clinical grades and glioma cell lines. We observed that SECTM1 was highly expressed in glioma and found that it was consistent with the analysis of bio information. SECTM1 expression was significantly correlated with survival. However, the role and mechanism of SECTM1 in GBM remain unclear. Subsequently, lentivirus knocked down the expression of SECTM1 in glioblastoma cell lines U87 MG and U251 MG. *In vitro* experiments have shown that knockdown SECTM1 expression can significantly inhibit the proliferation, invasion and migration of glioma cells, and *in vivo* experiments have proved that SECTM1 low expression has an inhibitory effect on the growth of glioma. The TGFβ pathway is activated in GBM and promotes glioma cell growth^15^. The expression of SECTM1 was significantly correlated with the TGFβ pathway by CGGA database analysis, and the correlation between TGFβ1 and the SECTM1 gene was high. WB assay showed that the expression of TGF-β1, TGF-βR2, p-Smad2 and p-Smad3 related proteins in transforming growth TGF-β pathway was decreased with SECTM1 down-regulation. It is suggested that SECTM1 may promote GBM invasion and EMT-like process by regulating the TGF-β1/Smad signaling pathway. These results indicate that SECTM1 may be a potential therapeutic target for glioblastoma.

## Materials and Methods

### Bioinformatics analysis

We used CGGA (http://www.cgga.org.cn) and TCGA data (https://www.cancergenome.nih.gov) to download the glioma gene expression data and clinical data. After the screening, we obtained 817 glioma patients (CGGA dataset) and 658 glioma patients (TCGA dataset). Using multiple R packages, including ggplot2, pROC, pheatmap, corrgram, circlize, Genomic Variation Analysis (GSVA), and survival analysis, the differentially expressed genes (DEGs) in the high SECTM1 and low SECTM1 groups were identified using an empirical Bayesian approach in the limma R package. The difference in tumor marker pathways between different groups was analyzed by "GSVA" R packet enrichment analysis. The "Hallmarks" gene set was downloaded from the MSigDB database for GSVA enrichment analysis. The cutoff value is set to p.value < 0.05, | Log2FC | > 1. GO and KEGG enrichment analysis of DEGs was performed using clusterProfiler in the R package.

### Sample collection

In this study, we collected tumor tissues from patients with gliomas admitted to Yijishan Hospital, the first affiliated hospital of the Wannan Medical College in recent years. Including 6 cases of low-grade gliomas (WHO grade Ⅰ and Ⅱ) and 6 cases of high-grade gliomas (WHO grade Ⅲ and Ⅳ). The study was approved by the Ethics Committee of Yijishan Hospital, the first affiliated hospital of the Wannan Medical College and Zhongda Hospital, Southeast University.

### Cell culture

The human embryonic kidney 293T (HEK 293T) cells, human glioblastoma cell line U87 MG, U251 MG, LN229, KNS81, and human normal astrocyte cell line (HA) were obtained from the Chinese Academy of Sciences Cell Bank (Shanghai, China). All cells were conventionally cultured in Dulbecco's Modified Eagle Medium (DMEM) (KGM12800-500, KeyGen Biotech, Nanjing, China) supplemented with 10% fetal bovine serum (FBS, F8318-500ML, Sigma-Aldrich) in a humidified incubator containing 5% CO_2_ and 95% air at 37°C.

### Screening of SECTM1 stable knockdown cell lines

SECTM1 stable knockdown cell lines were obtained by screening after lentivirus transfection. Briefly, shRNA plasmid (plko.1 backbone vector) or control vector plasmid followed the lentiviral transduction protocol^16^, transduced HEK 293T cells to obtain SECTM1 knockdown lentivirus, and then transfected the lentivirus into target cells U87 MG and U251 MG, to obtain SECTM1 knockdown cell lines of U87 MG and U251 MG cells (shNC, shSECTM1). The stably transfected cell lines were obtained by adding 2 mg/ml Puromycin (ST551, Beyotime Technology) into lentivirus-infected cells for two weeks. The shRNA sequences were as follows: sh- SECTM1-1: 5′- CACCAGAGAAATAACAGACAA -3′, sh-SECTM1-2: 5′- GTGTTCAAACCCTCACCACTT -3′.

### Cell proliferation assay (CCK8 assay)

The effect of SECTM1 knockdown on the proliferation of cells (U87 MG, U251 MG) was detected by cell counting kit-8 (CCK-8). U87 MG or U251 MG stable cell lines were seeded in 96-well cell culture plates at 3000 cells/well density. After culturing the cells for 2 hours (cells were adherent), 10 µL of CCK-8 solution (B1099, Bioogenetech, Hefei, China) was added to each well and incubated for 1 hour, then the absorbance at 450nm was measured using a microplate reader (BioTek). Cells were subsequently assayed for CCK-8 every 24 hours for five days, and cell proliferation curves were drawn.

### Cell cycle detection

The effect of SECTM1 on the cell cycle was detected by flow cytometry. Stably transfected U87 MG or U251 MG cell lines were inoculated into 6-well cell culture plates at a density of 5×10^5^ cells/well. After 24 h of culture, cells were collected in 1.5 mL 75% ethanol and fixed overnight at -20°C. The cells were treated with the cell cycle detection kit (KGA512, Keygen). Flow cytometry (Beckman, Navios) and data were analyzed using Modfit software.

### Cell migration and invasion assay (transwell assay)

The effect of SECTM1 knockdown on the migration capacity of cells (U87 MG, U251 MG) is determined by transwell assay using a chamber with transwell inserts in 24-well plates with a pore size of 8 µm. 5×10^4^ cells (U87 MG or U251 MG stable cell lines) / well were suspended in 200 µL serum-free DMEM medium and seeded in the upper chamber. 800 µL DMEM containing 10% FBS was added to the lower chamber. For the invasion assay, the upper chamber was treated with Matrigel (1:3 dilution, BD Bioscience, CA, USA) before cell seeding. After 48 h of incubation, cells are fixed with 4% paraformaldehyde **(**PFA**)** for 20 min and stained with 1% crystal violet staining solution for 30 min, followed by cleaning and observed and photographed under an upright microscope (Carl Zeiss Axio Scope Al, Germany). The number of migrated cells was analyzed using Image-J software.

### EdU (5-Ethynyl-2'-deoxyuridine) detected cell proliferation

The effect of SECTM1 knockdown on cell (U87 MG, U251 MG) proliferation was detected by EdU. U87 MG or U251 MG stable cell lines are seeded in 6-well cell culture plates at a density of 5×10^5^ cells/well. After 24 h of culture, stain by kFluor488-EdU cell proliferation detection kit (KGA331, Keygen Biotech, Nanjing, China), with an EdU working concentration of 50 µM, and cells were incubated for 2 h after the addition of EdU. Observed and photographed under a fluorescence microscope (Carl Zeiss Axio Scope Al, Germany). Cell proliferation percentage is measured using Image-J software.

### Wound healing assay (cell scratching assay)

The effect of SECTM1 knockdown on the migration capacity of cells (U251 MG) was verified by wound healing experiments. U251 MG stable cell lines are seeded in 6-well cell culture plates at a density of 5×10^5^ cells/well. Once the confluence of cells reaches 90-100%, the tip of the sterile pipette is used to scratch the monolayer cells in a straight line to simulate the wound. The cells were then washed using PBS to remove cell debris. Serum-free DMEM medium was added to the culture, photographs of the scratch wound were taken using an inverted microscope (Carl Zeiss Axio Scope Al, Germany) at 0 h and 24 h of incubation, and the scratch area was measured with Image-J software.

### Xenograft mouse model

The Balb/c nude mice (6-8w, 18-22 g) were obtained from GemPharmatech (Nanjing, China). The mice were housed in an SPF-level vivarium with free access to food and water. All animal studies were conducted in accordance with the guidelines of the National Regulations on the Care and Use of Laboratory Animals in China and approved by the Animal Care and Use Committee (IACUC) of Wannan Medical College. All procedures are following the provisions of the Agency's Animal Care and Ethics Committee. In an *in vivo* animal study, the constructed U87 MG cells (5x10^6^) stably expressing shNC or shSECTM1 were injected subcutaneously into BALB/c nude mice. Tumor size and body weight were measured every 2 days after one week of injection, and tumor volume was calculated as length×width^2^/2. The tumors were collected on day 23 post-injection and made into frozen sections for immunohistochemistry.

### Mouse model of intracranial tumor formation *in situ*

Referring to the previous method 17, it was briefly described as follows: U87 MG-LUC-NC, U87 MG-LUC-sh1, U87 MG-LUC-sh2 cells were suspended in PBS at a density of 3× 10^7^/ml. Subsequently, the mice were anesthetized and fixed, and the skin was cut along the midline of the head of the mice for about 1 cm, exposing the cranium and fontanelle, a hole was drilled at a point of 1 mm from the fontanelle and the stereotactic apparatus was then adjusted to make sure that the micro-syringe was located at the puncture point and 10 μl of the cell suspension was aspirated. The needle was slowly inserted vertically for 4 mm, then backed up for 1 mm, and finally injected slowly at a rate of 2 μl/min, and then gradually withdrawn after keeping the needle in the original position for 2 min. One week after tumor formation, the fluorescence intensity of luciferase in the brain of nude mice was detected by an animal *in vivo* imaging system (IVIS Lumina LT, PerkinElmer) every 5 days (n = 4/group). Two weeks after imaging, the mice were executed and the brains were removed to make frozen sections for subsequent experiments.

### H&E stained

Frozen tissue sections of mouse tumors were fixed with 4% paraformaldehyde for 15 minutes and rinsed three times with PBS. The Hematoxylin staining solution was stained for 5 min, and the excess staining solution was removed by rinsing in water. The staining solution was differentiated in differentiation solution for the 20s, treated with the blue promoting solution for 1min, and washed with water for 1min. Eosin was stained for 1 min, washed in water for about the 30s, and dehydrated through gradient alcohol, 75% ethanol for 10s, 85% ethanol for 10s, 95% ethanol for 10s, and absolute ethanol for 10s. Xylene is transparent for 5 minutes. Seal the sheet with neutral resin. Observations were made under a positive microscope (Carl Zeiss Axio Microscope Al, Germany).

### Statistical Analysis

GraphPad Software was used to analyze all statistics of this study. Statistical differences between groups were analyzed by a two-tailed Student t-test. Experimental data were expressed as mean ± standard deviation (error bars). *P < 0.05 indicates a statistically significant difference, ****P < 0.0001 indicates a significant difference.

## Results

### SECTM1 is highly expressed in glioblastoma

**Figure 1:**
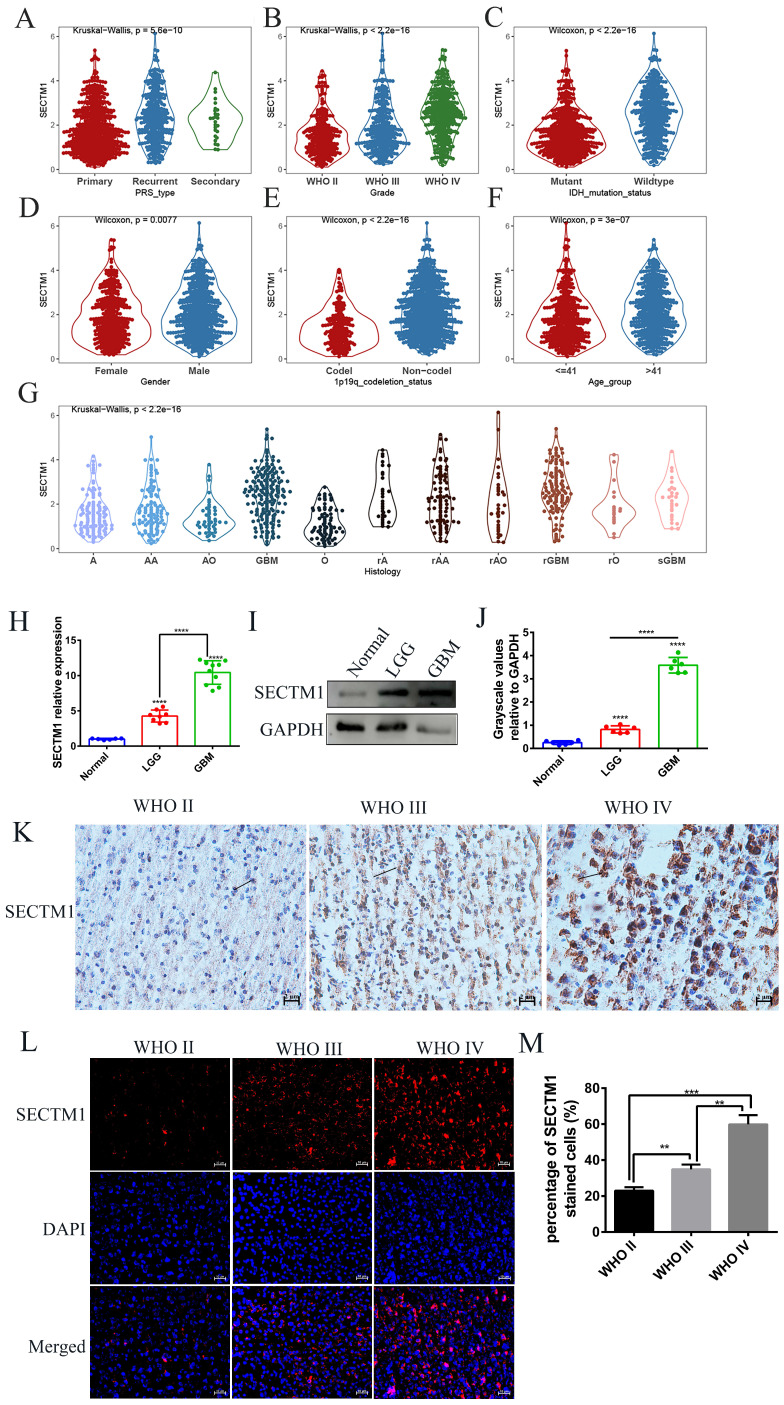
Expression of SECTM1 in data sets and clinical tissue samples. Kruskal-Wallis analyzed the relative expression difference of SECTM1 in patients with primary GBM, recurrent GBM and secondary GBM in the CGGA database (A). Kruskal-Wallis analyzed the relative expression difference of SECTM1 in Grade II, III and IV glioma patients in the CGGA database (B). C-F was the relative expression difference of SECTM1 in IDH mutation and wild-type patients (C), different sex patients (D), 1p19q deletion and non-deletion patients (E), and patients younger than 41 years old and older than 41 years old (F). In the CGGA database, Kruskal-Wallis analyzed the relative expression difference of SECTM1 in different histological grades of GBM (G), H-J was RT-qPCR (H) or western blot (I-J) to detect the relative expression difference of SECTM1 in normal brain tissue, LGG tissue and GBM tissue. K-M was IHC staining (K) and immunofluorescence staining (L-M) to detect the relative expression difference of SECTM1 in glioma II, III and IV patients, respectively, with scales of 2μm and 10μm. All data were analyzed by T-test for P-values as the mean ±SEM of three independent experiments. ** P <0.01; *** P < 0.001; **** P < 0.0001.

First, we analyzed the difference in SECTM1 expression between gliomas and the normal group by the GTEX data set (http://gepia.cancer-pku.cn/detail.php), which showed that SECTM1 was highly expressed in GBM compared with the normal group (Figure S1A). Then, we analyzed the expression of SECTM1 in the CGGA dataset. The results showed that the expression of SECTM1 in recurrent or secondary gliomas was significantly higher than that in primary gliomas (Table 1, Figure 1A), and the expression of SECTM1 was positively correlated with the malignancy degree of gliomas. The expression of SECTM1 in WHO grade Ⅳ gliomas was significantly higher than that in WHO grade Ⅱ and Ⅲ gliomas (Table 1, Figure 1B). The expression level of SECTM1 in the IDH wild type glioma was higher than that of the IDH mutant type (Table 1, Figure 1C). The expression level of SECTM1 in male patients was higher than that in female patients (Table 1, Figure 1D). The expression of SECTM1 in the lp/19q co-deletion group was higher than that in the non-deletion group (Table 1, Figure 1E). SECTM1 expression was higher in patients older than 41 years than in patients younger than 41 years (Table 1, Figure 1F). The expression of SECTM1 in different glioma tissue types showed statistically significant differences (Table 1, Figure 1G).

The high expression of SECTM1 in GBM was also confirmed in clinical tissue samples (Figure 1H-M). qPCR results showed that SECTM1 mRNA was highly expressed in glioma tissues (Figure 1H). At the protein level, western blot (Figure 1I-J), immunohistostaining (Figure 1K, Figure S1B) and immunofluorescence staining (Figure 1L-M) experiments showed that compared with normal brain tissue, SECTM1 was highly expressed in glioma tissue, and the higher the malignant degree of glioma, the higher the expression of SECTM1.

### High expression of SECTM1 is associated with poor survival and prognosis in glioma patients

**Figure 2:**
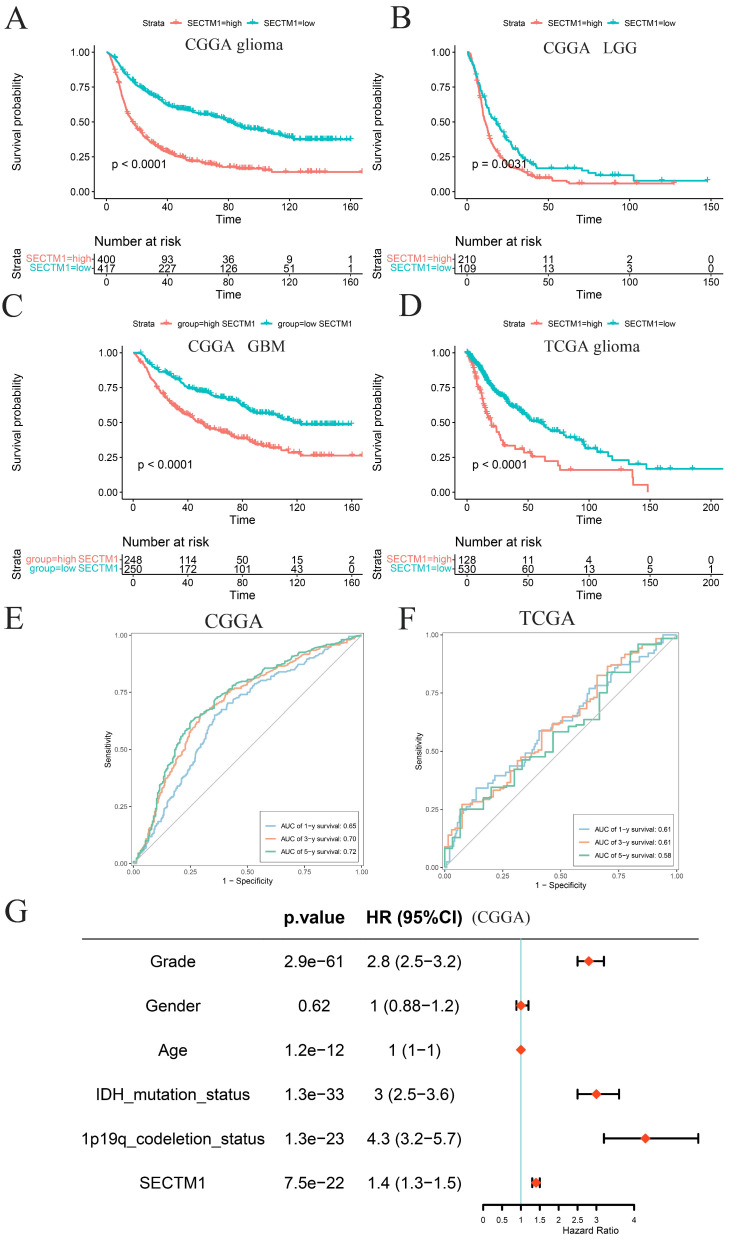
The expression of SECTM1 in glioma is associated with the survival and prognosis of patients. A-C refers to the correlation between the expression of SECTM1 and the survival of glioma (A), LGG (B) and GBM (C) patients in the CGGA database by Kaplan-Meier analysis, while the expression of SECTM1 in the TCGA database by Kaplan-Meier analysis and the survival of glioma patients (D). E-F was the ROC curve of prognostic models in CGGA database (E) and TGGA database (F) to analyze the correlation between SECTM1 and the prognosis of glioma patients, and the expression of SECTM1 in CGGA database to analyze the correlation with glioma grade, gender, age, IDH mutation, 1p19q chromosome deletion and other factors (G).

According to the median expression of SECTM1, glioma patients were divided into a high-expression group and a low-expression group. Survival analysis of glioma patients in the CGGA data set showed that the overall survival (OS) of patients with high SECTM1 expression was significantly shorter than that of patients with low SECTM1 expression (Figure 2A). In low-grade glioma (Figure 2B) and high-grade glioma (Figure 2C), patients with the high SECTM1 expression group had significantly shorter OS than those with the low expression group. In the TCGA glioma patient data set, the OS of the SECTM1 group with high expression was significantly shorter than that of the group with low expression (Figure 2D), which was consistent with the analysis of the CGGA dataset. ROC curves of prognostic models analyzed in CGGA and TCGA glioma patient data sets showed that high expression of SECTM1 was closely associated with poor prognosis of glioma patients (Figure 2E-F). In addition, the high expression of SECTM1 was closely related to glioma grade, IDH mutation and 1p19q deletion (Figure 2G). In summary, the expression of SECTM1 is significantly correlated with the survival of glioma patients.

### SECTM1 is highly expressed in glioblastoma cell lines

In addition, we also found that the expression of SECTM1 in GBM cell lines was consistent with clinical tissue samples, and qPCR results showed that compared with human astrocytes (HA), the expression level of SECTM1 mRNA in human GBM cell lines (U87 MG, U251 MG, LN229, KNS81) was significantly increased (Figure S2A). At the protein level, experiments on western blot (Figure S2B-C) and Immunofluorescence staining (Figure S2D-E) showed that SECTM1 was highly expressed in GBM cell lines compared with normal cell HA.

### Knockdown of SECTM1 inhibited GBM cell proliferation

**Figure 3:**
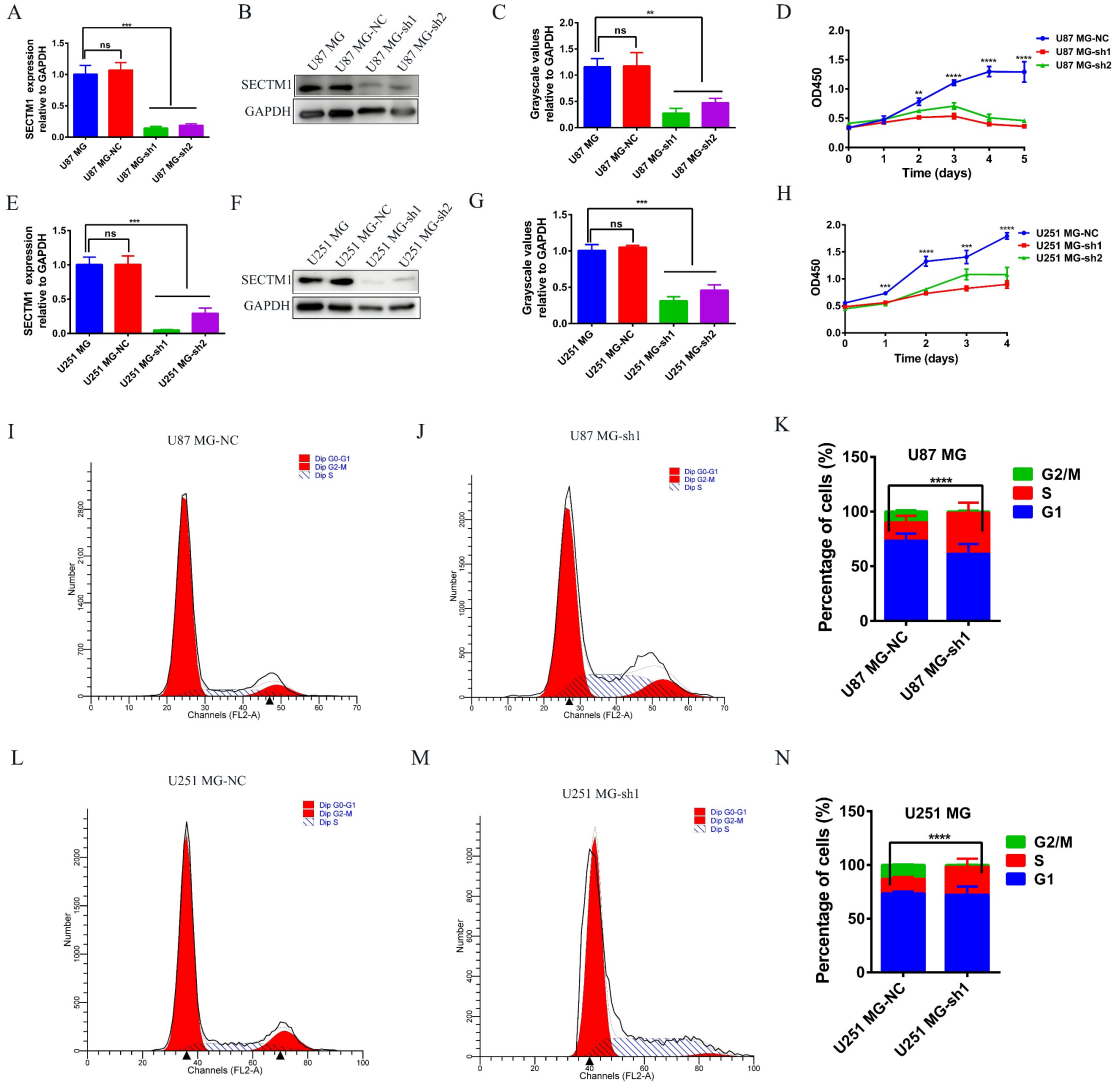
The expression of SECTM1 was knocked down and the effect of SECTM1 on GBM cell proliferation was detected. shRNA inhibition of SECTM1 mRNA and protein expression in U87 MG (A-C) and U251 MG (E-G) were detected by RT-qPCR or western blotting, and the gray value statistics were obtained. CCK-8 was used to detect the effect of SECTM1 knockdown on the proliferation of U87 MG (D) and U251 MG (H). Image and statistical analysis of the effect of SECTM1 knockdown on U87 MG (I-K) and U251 MG (L-N) cell cycle by flow cytometry. All data were analyzed by T-test for P-values as the mean ±SEM of three independent experiments. *P < 0.05; ** P <0.01; *** P < 0.001.

To determine the role of SECTM1 in GBM, we constructed shRNAs targeting SECTM1 (SECTM1-sh1, SECTM1-sh2) and negative control shNC, knocked down the expression of SECTM1 in GBM cells (U251 MG and U87 MG cells), RT-qPCR and immunoblot analysis detected knockdown effects (Figures 3A-C, E-G). qPCR (Figures 3A and E). and WB experiments (Figures 3B-C, F-G) showed that SECTM1-sh1, and SECTM1-sh2 knockdown SECTM1 in both U251 MG and U87 MG cells compared with shNC, with higher knockdown efficiency of SECTM1-sh1 (Figures 3A-C, E-G).

**Figure 4:**
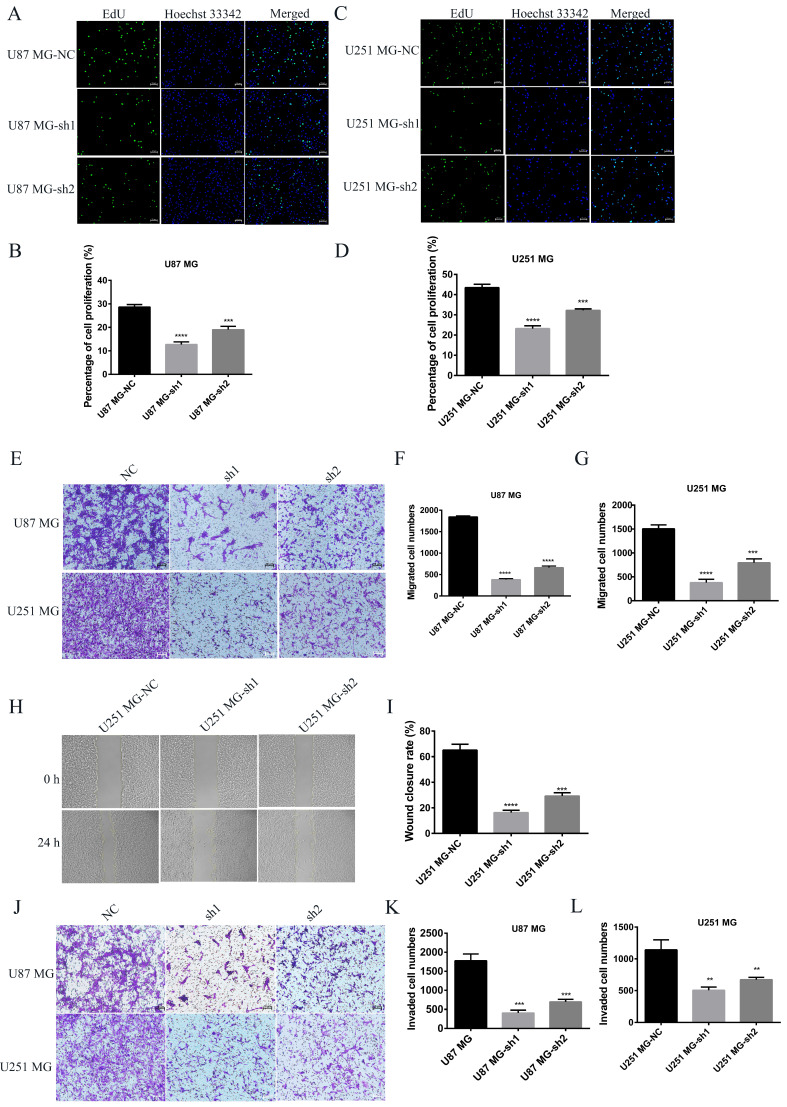
The effect of silencing SECTM1 expression on the proliferation, migration and invasion of GBM cells was detected. Fluorescence image of the effect of EdU detection on proliferation of U87 MG (A-B) and U251 MG (C-D) cells after SECTM1 knockdown expression, scale, 10 μm. Representative images (E) and statistical (F-G) analysis of cell migration ability after SECTM1 knockdown in U87 MG and U251 MG, scale, 10μm. Representative image (H) and relative migration area (I) of cell wound healing assay after SECTM1 knockdown in U251 MG. Representative image of cell invasion assay after SECTM1 knockdown in U87 MG and U251 MG (J) and statistical number of cells invaded (K-L). The data were expressed as the mean value of the three experiments ±SEM, ** P <0.01; *** P < 0.001; **** P < 0.0001.

CCK-8 experiment showed that SECTM1 knockdown significantly reduced the proliferation of U87 MG and U251 MG cells (Figure 3 D and H), and the higher the SECTM1 knockdown degree, the stronger the inhibition of cell proliferation. These data indicated that down-regulation of SECTM1 inhibited cell proliferation. In addition, the cell cycle distribution was analyzed by flow cytometry. We found that in U87 MG (Figure 3I-K) and U251 MG cell lines (Figure 3L-N) SECTM1 down-regulated cells were significantly enriched in the S phase, and cell cycle arrest occurred in the S phase (Figure 3I-N). This was also consistent with KEGG enrichment analysis (Figure S3A), and SECTM1 was a G2/M phase checkpoint. We further verified by EdU experiments that the knockdown of SECTM1 significantly reduced the proliferation of U87 MG (Figure 4A-B) and U251 MG cells (Figure 4C-D), and the higher the SECTM1 knockdown degree, the stronger the inhibition of cell proliferation.

### Knockdown of SECTM1 inhibited GBM cell migration and invasion

To further determine whether SECTM1 plays a role in the progression of GBM, the effect of SECTM1 on cell migration and invasion was verified by wound healing and transwell assays. Compared with the control group, the migration ability of U87 MG and U251 MG cells was significantly inhibited after SECTM1 knockdown (Figure 4E-I). The invasion ability of cells was also inhibited (Figure 4J-L), and the higher the SECTM1 knockdown degree, the stronger the inhibition of cell migration and invasion ability.

### SECTM1 regulates GBM invasion through the TGF-β1/Smad signaling pathway and participates in the EMT-like process of GBM cells

**Figure 5:**
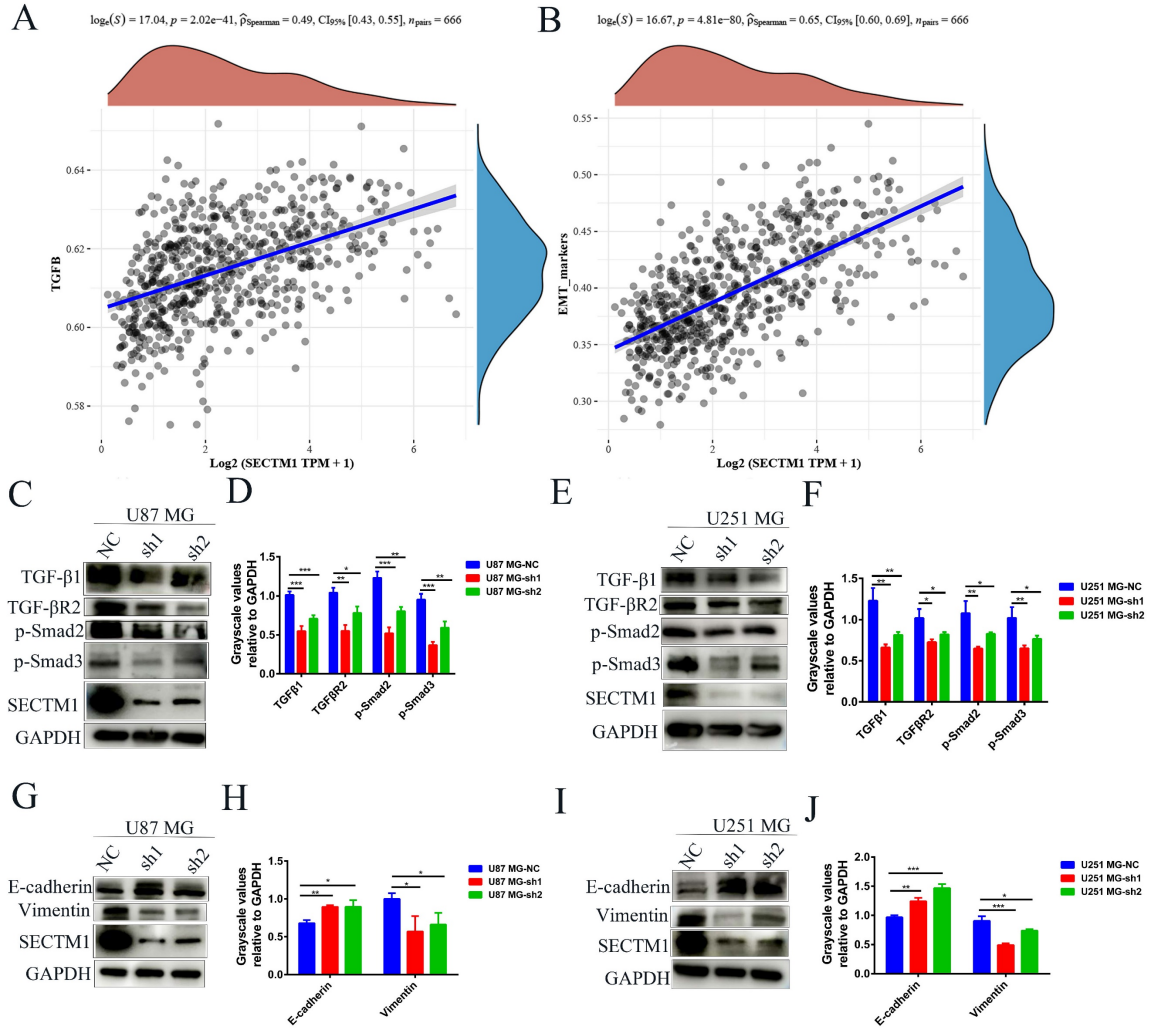
SECTM1 regulates glioma migration and invasive phenotype through TGF-β1/Smad pathway and SECTM1 knockdown inhibits the EMT-like process in glioma. The correlation between SECTM1 gene and TGFβ pathway (A) and EMT marker (B) in glioma was analyzed in TCGA. (C-F) Western blotting showed the changes of downstream genes of TGF-β1/Smad pathway after SECTM1 knockdown in U87 MG cells (C, D) and U251 MG (E, F), where D and F were the quantitative gray values of WB experiment. All data were presented as mean ± SD (three independent experiments). * P < 0.05; * * P < 0.01; * * * P < 0.001.

To explore the molecular mechanism of the SECTM1 effect on GBM, we analyzed the correlation between genes and pathways in TCGA and CGGA databases. The results showed that the TGF-β signaling pathway was positively correlated with the expression of SECTM1 (Figure 5A, Figure S3A). The CGGA database was used to detect the relationship between TGF-β signaling pathway genes and SECTM1, and it was found that SECTM1 had a strong positive correlation with TGF-β1 (Figure S3B). qPCR detected significant downregulation of TGFβ1 expression in U87MG (Figure S3C) and U251 MG (Figure S3D) cells after SECTM1 knockdown (Figure S3C-D). We further detected the downstream molecules of the TGF-β1/Smad signaling pathway by WB, and found that the expression levels of TGF-β1, TGF-βR2, p-Smad2 and p-Smad3 in U87MG (Figure 5C-D) and U251 MG cells (Figure 5E-F) were significantly down-regulated after SECTM1 knockdown (Figure 5C-F). These results suggest that SECTM1 can regulate the proliferation, invasion and migration of GBM cells through TGF-β1/Smad signaling pathway.

We also analyzed the correlation between genes and EMT in the TCGA database and found that EMT markers were significantly correlated with the expression of SECTM1 (Figure 5B), indicating that SECTM1 was involved in the EMT-like process. We then further detected the expression of EMT-related markers by WB, and found that after SECTM1 knockdown, E-cadherin expression increased and vimentin expression decreased in U87MG (Figure 5G-H) and U251 MG cells (Figure 5I-J). These results suggest that SECTM1 is involved in the EMT-like process of GBM cells, which may also be regulated by the TGF-β1/Smad signaling pathway.

**Figure 6:**
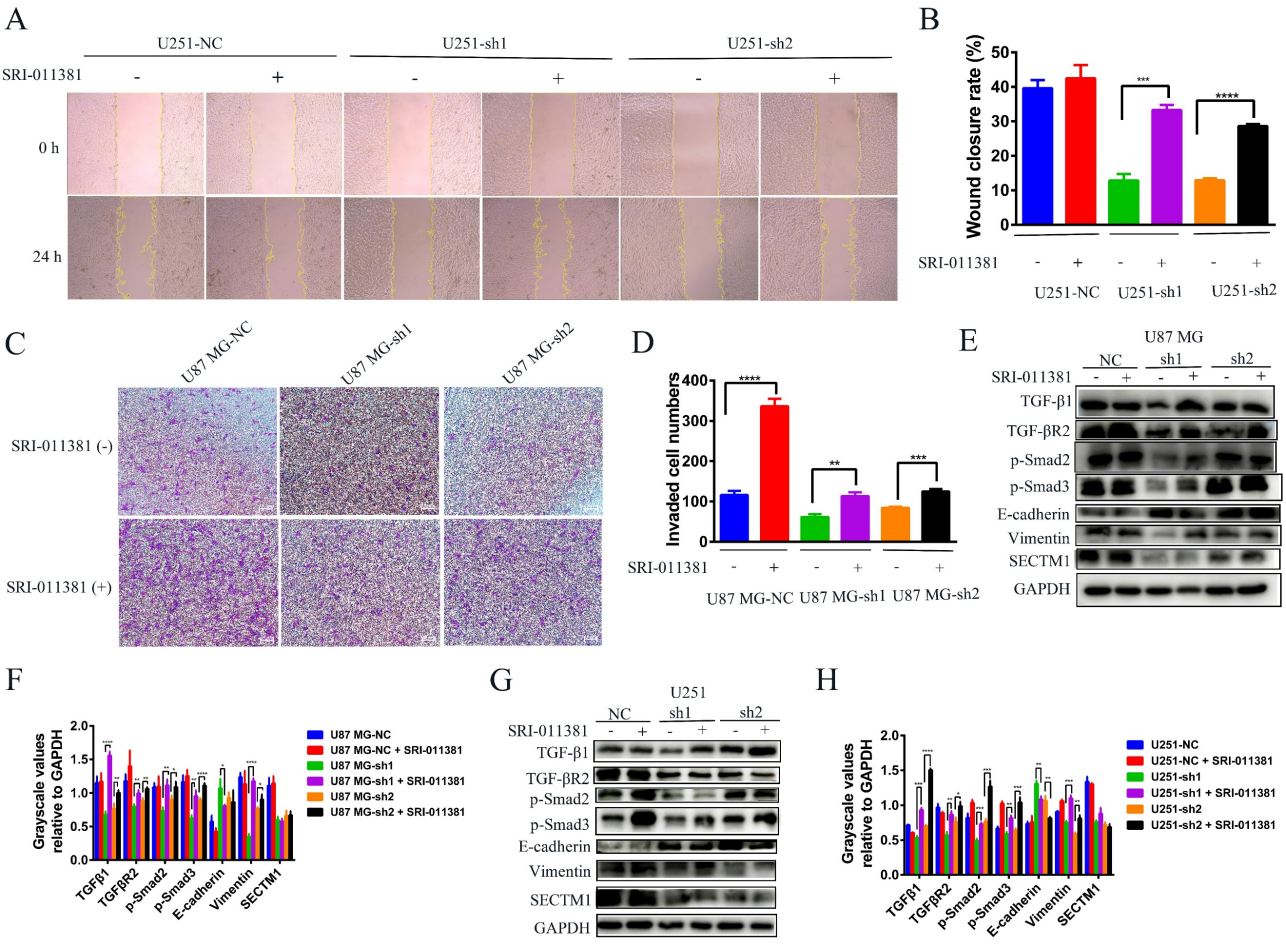
Wound healing assay, invasion assay and WB assay to verify the regulation of TGF-β1/Smad signaling pathway on the involvement of SECTM1 in the EMT process. (A) Representative images of cellular wound healing assay in U251 cells (U251-NC, U251-sh1, U251-sh2) after 24h of treatment or no treatment with TGF-β1 agonist SRI-011381. (B) Relative migration area of wound healing assay. (C) Representative images of cell invasion after 24h of TGF-β1 agonist SRI-011381 treatment or no treatment of U87 MG cells (U87 MG-NC, U87 MG-sh1, U87 MG-sh2). (D) The number of invaded cells counted. (E) WB experimental images of TGFβ pathway and EMT-related markers after 24h of TGF-β1 agonist SRI-011381 treatment or no treatment of U87 MG cells (U87 MG-NC, U87 MG-sh1, U87 MG-sh2). (F) Statistical analysis of the gray-scale quantitative values of WB experiments in the E-figure. (G) WB experimental images of TGFβ pathway and EMT-related markers in U251 cells (U251-NC, U251-sh1, U251-sh2) after 24h of TGF-β1 agonist SRI-011381 treatment or no treatment. (H) Statistical analysis plot of the gray-scale quantitative values of WB experiments in G Fig. All data were analyzed by t-test and shown as mean ± SD (three independent experiments), *P < 0.05; ** P <0.01; *** P < 0.001; **** P < 0.0001.

Next, we verified whether the EMT-like process involved by SECTM1 was regulated by the TGF-β1/Smad signaling pathway. We used the TGF-β1 agonist SRI-011381 hydrochloride (10 μM, T5129, Topscience) to treat U87 MG cells (U87 MG-NC, U87 MG-sh1, U87 MG-sh2) and U251 cells (U251-NC, U251-sh1 U251 cells (U251-NC, U251-sh1, U251-sh2) for 24 h. The regulation of EMT-like processes by the TGF-β1/Smad signaling pathway was verified by wound healing, invasion, and WB assay. Firstly, the wound healing assay showed that after SRI-011381 treatment, the migration ability of U251-sh1 and U251-sh2 cells was significantly enhanced, which almost counteracted the inhibitory effect of SECTM1 knockdown (Figure 6A-B). The invasion assay also demonstrated that relative to the SECTM1 knockdown group, SRI-011381-treated U87 MG- sh1 and U87 MG -sh2 cells were significantly enhanced (Figure 6C-D). The results indicated that with the activation of the TGF-β1/Smad signaling pathway, the invasive ability of cells after SECTM1 knockdown was also enhanced. Subsequently, the regulation of the TGF-β1/Smad signaling pathway on the involvement of SECTM1 in the EMT-like process was verified at the protein level. The results showed that, both in the U87 MG cells and the U251 cells, after the treatment of SRI-011381, the expression of TGF-β1 in the SECTM1 knockdown group was significantly elevated. The TGF-β1/Smad signaling pathway was activated (Figure 6E-H). Meanwhile, the expression of EMT marker E-cadherin was decreased and Vimentin expression was increased, indicating that the activation of the TGF-β1/Smad signaling pathway promoted the EMT process and thus enhanced the cell invasion ability after SECTM1 knockdown (Figure 6E-H). The above results indicated that the TGF-β1/Smad signaling pathway regulated the EMT-like process involved in SECTM1.

### Knockdown of SECTM1 inhibited tumor growth in mice

**Figure 7:**
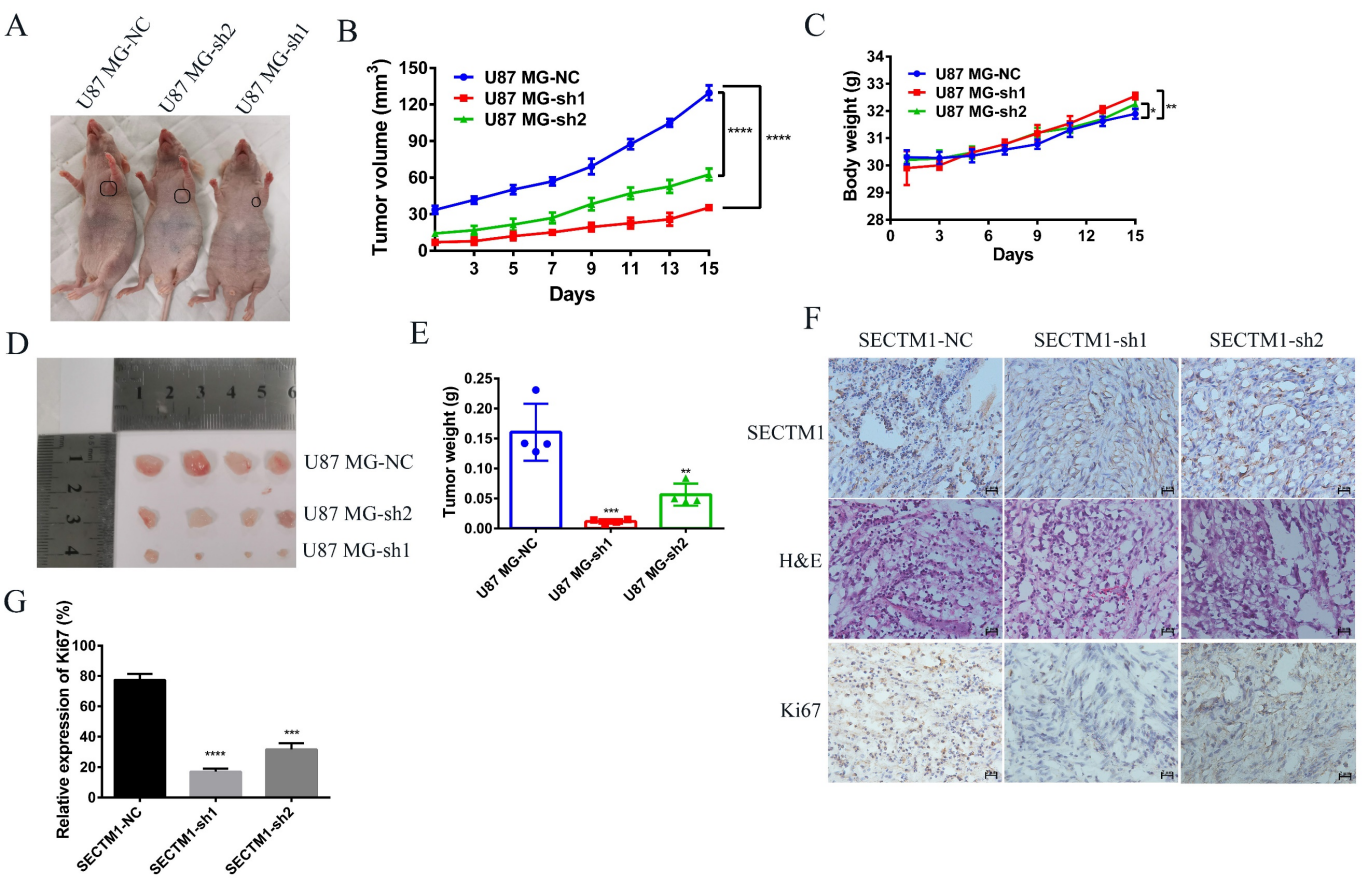
Effect of SECTM1 knockdown in U87 MG on tumorigenicity in mice. Image of BALB/c nude mice after subcutaneous neoplasia (A). Statistical analysis of tumor volume (B) and body weight (C) (n = 4) in nude mice measured every 2 days. Image of mouse subcutaneous tumor removal (D) and statistical analysis of tumor weight (E). Expression image of SECTM1 and proliferative marker Ki-67 by H&E staining and IHC analysis in control or shSECTM1 xenograft tumor tissue (F), scale, 2 µm. (G) Relative expression percentage of Ki67 in IHC. The data represent the mean ±SEM of the T-test analysis through three independent experiments, *P < 0.05; ** P <0.01; *** P < 0.001; **** P < 0.0001.

Next, we used a tumor model of subcutaneous xenografted U87 MG cells (U87 MG-NC, U87 MG-sh1, U87 MG-sh2) to investigate whether the above *in vitro* findings apply to animals. We found that compared with the negative control SECTM1-NC, the tumor with SECTM1 knockdown was significantly smaller and the tumor growth was slower (Figure 7A-E), and the higher the degree of SECTM1 knockdown, the slower the tumor growth. The expression of SECTM1 is effectively suppressed in xenograft tumors (Figure 7F, Figure S4A). Tissue sections are then collected for H&E staining and immunohistochemical staining. The results showed that the proliferation marker Ki67 was significantly reduced in U87-cell-derived tumor tissue with SECTM1 knockdown (Figure 7F-G, Figure S4B-C). These results indicate that SECTM1 knockdown can dramatically inhibit tumor growth in mice, and SECTM1 may be a potential target for glioblastoma treatment.

### Knockdown of SECTM1 inhibits tumor growth and invasion *in situ* tumor-bearing mice

**Figure 8:**
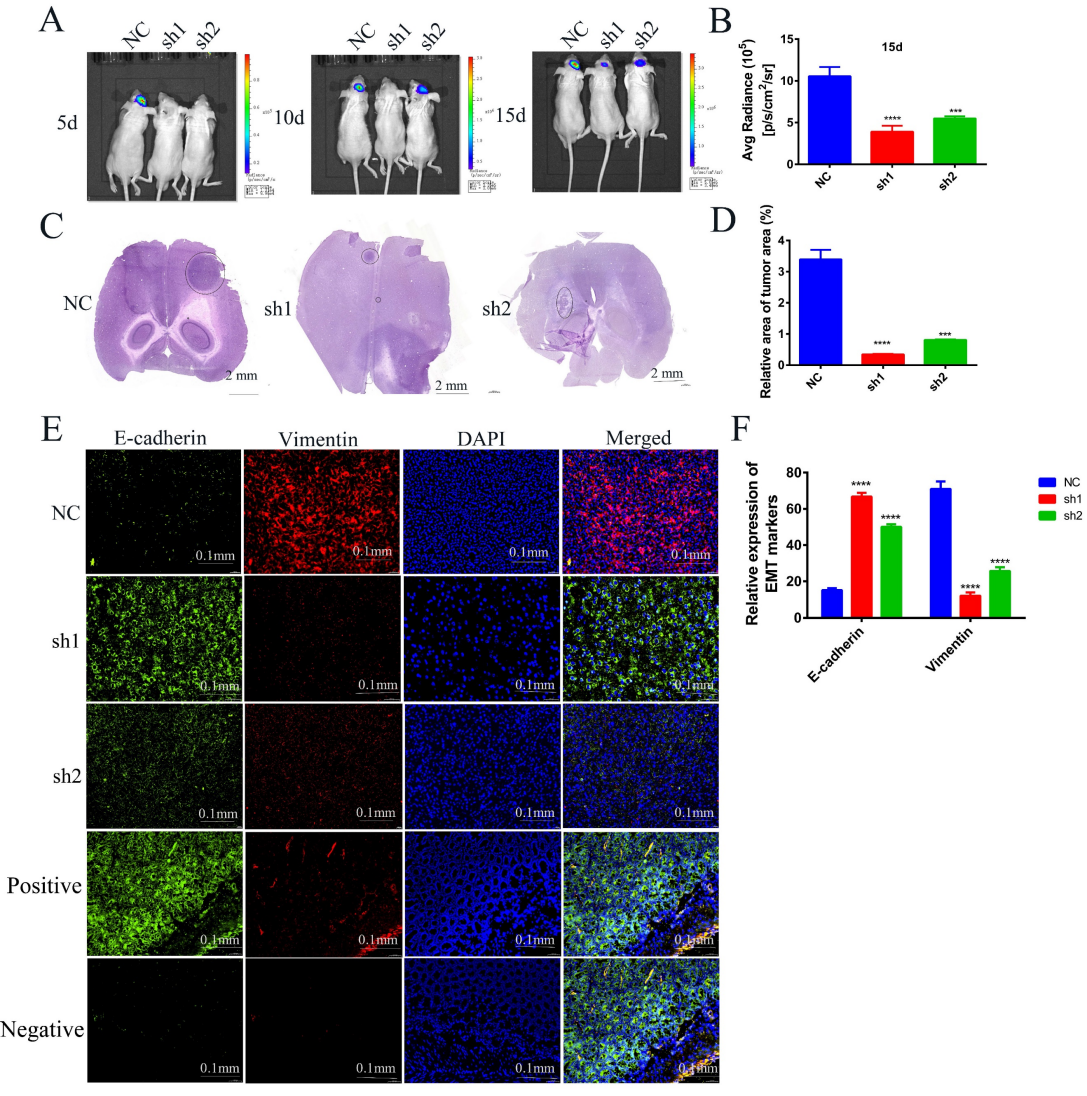
Effects of knockdown of SECTM1 in U87 MG on *in situ* tumorigenesis in mice. (A) *In vivo* bioluminescence imaging of mice was detected every 5 days after 7 days of *in situ* tumorigenesis of different cell groups (n=4). (B) The intensity of luciferase bioluminescence in mouse brain *in situ* glioma tissues. (C) HE staining images of frozen sections of *in situ* glioma mouse brain tissues from different groups after 15 days of imaging, scale bar of 2 mm. (D) Quantitative analysis of relative tumor volume in HE staining. (E) EMT markers immunofluorescence staining images of frozen brain tissue sections of mice with *in situ* loaded tumors from different groups. Mouse colon tissue as a positive control for E-cadherin antibody and isotype IgG as a negative control. (F) Quantitative analysis of EMT markers immunofluorescence staining results. The data represent the mean ±SEM of the T-test analysis through three independent experiments, *P < 0.05; ** P <0.01; *** P < 0.001; **** P < 0.0001.

To further investigate the effects of SECTM1 knockdown on the tumorigenicity of U87 MG cells and on tumor growth and invasion, we used U87 MG-LUC cells (U87 MG-LUC-NC, U87 MG- LUC-sh1, U87 MG- LUC-sh2) to *in situ* tumorigenicity in nude mice and, at different time points (5 days, 10 days, 15 days) detected the luminescence range and intensity of the tumors in the mouse brain. The results showed that tumors were detected in the NC group mice at the first imaging and the sh1 and sh2 groups mice because the fluorescence intensity was too low to detect tumors (Figure 8A, Figure S5A). With the increase of tumorigenesis time, the brains of mice in the NC Group showed an increasing range of fluorescence, which indicated a gradual invasion to the peritumor, and the fluorescence intensity was also increasing, indicating that the tumor cells were proliferating (Figure 8A-B, Figure S5A). In contrast, mice in the sh1 group could not detect the tumor until the third imaging and the fluorescence intensity was weak, and mice in the sh2 group showed little expansion of the tumor extent in the third imaging compared with the second imaging (Figure 8A-B, Figure S5A). The results of *in vivo* imaging indicated that SECTM1 knockdown inhibited tumorigenicity of tumor cells and tumor proliferation and invasion.

In order to observe the size of mouse brain tumors more intuitively, we made frozen sections of mouse brains and performed HE staining. The staining of the tumor tissue was deepened and the nuclei were heterogeneous. The results showed that compared with the brain tumor area of mice in the NC group, the brain tumor area of mice in the sh1 and sh2 groups was very small. The smallest tumor was found in the sh1 group (Figure 8C-D, Figure S5B), and this result intuitively demonstrated that the SECTM1 knockdown inhibited the proliferation of the tumors.

To verify whether SECTM1 was involved in the EMT process *in vivo*, we performed immunofluorescence staining for EMT markers (E-cadherin: green, Vimentin: red) on brain-frozen sections of *in situ* tumor-bearing mice and tumor tissue frozen sections of subcutaneous tumor-bearing mice, respectively. Staining of brain-frozen sections from *in situ* tumor-bearing mice showed increased E-cadherin expression and decreased vimentin expression in the SECTM1 knockdown group compared with the NC group (Figure 8E-F, Figure S6). E-adhesins are expressed on the cell surface of most epithelial tissues and are abundantly expressed in the epithelial cells of mouse colonic tissues^18^-^20^. Therefore, we used mouse colon tissue as a positive control for E-cadherin antibody and isotype IgG antibody as a negative control. The staining results showed that E-cadherin was highly expressed on the surface of epithelial cells in mouse colon tissue (Figure 8E). The results of positive and negative control showed that the specificity of the E-cadherin antibody was good. The same results were obtained from the frozen section staining results of tumor tissues of subcutaneous tumorigenic tumors (Figure S7). The results indicated that SECTM1 knockdown inhibited the EMT process, thereby suppressing tumor invasion.

## Discussion

GBM is one of the most refractory malignancies at present, and its treatment standard is postoperative chemotherapy and radiotherapy^21^, with a limited choice of chemotherapeutic agents to treat GBM, and the current chemotherapeutic agents are mainly temozolomide (TMZ). However, due to the existence of the blood-brain barrier (BBB), poor drug targeting, side effects, short biological half-life and other limitations, chemotherapeutic drugs are less effective^22^, ^23^. GBM also leads to poor treatment results due to complex tissue specificity and individual differences. Efforts have been made to find and develop new therapies for GBM, in which molecularly targeted therapies are one of the effective means to treat GBM^24^, ^25^.

SECTM1 is a -secreted protein belonging to the SECTM family, which plays a vital role in cell signal transduction and the hematopoietic/immune system^26^, ^27^. Several studies have reported that overexpression of SECTM1 is associated with melanoma, prostate cancer, breast cancer and some myeloid leukemia^10^, ^12^-^14^. In this study, we found that SECTM1 was highly expressed in glioma and significantly correlated with the survival of glioma patients. The role and mechanism of SECTM1 in glioma were elucidated for the first time.

In gliomas, the proportion of LGG and secondary GBM initially found is high, while the proportion of primary GBM is low^28^. According to the analysis of the CGGA database, SECTM1 is highly expressed in secondary and recurrent gliomas, indicating that SECTM1 may promote the proliferation, invasion and migration of GBM. This also suggests that SECTM1 may be used as a diagnostic marker. In addition, studies have shown that the presence of IDH mutations in gliomas significantly improves progression-free survival compared to IDH wild-type gliomas regardless of treatment^29^. IDH mutant gliomas are more sensitive to drug therapy and are not prone to drug resistance^30^. The expression level of IDH wild-type SECTM1 in gliomas is higher than that of IDH mutant. However, whether silting SECTM1 can enhance the sensitivity of drug therapy needs further experimental verification and further indicates that SECTM1 can be used as a potential target for drug therapy. 1p/19q chromosome co-deletion has been considered as a diagnostic and prognostic marker for oligodendroglioma since 1998^31^, and the high expression of SECTM1 in 1p/19q non-codel indicates that SECTM1 expression is significantly correlated with the survival and prognosis of glioma patients.

By regulating the expression of SECTM1 in glioma cell lines, we found that the proliferation, migration and invasiveness of U87MG and U251 MG glioma cells were significantly attenuated after the knockout of SECTM1. *In vivo* experiments also demonstrated that SECTM1 knockdown inhibited tumor growth. EMT is a common phenomenon in epithelial-derived malignant tumors and is involved in the invasion and treatment tolerance of malignant tumors^32^. Our experiments confirmed that SECTM1 regulates EMT-like processes in GBM cell lines, thereby promoting glioma cell invasion. Based on the analysis of TCGA and CGGA GBM databases, it was found that the TGF-β signaling pathway was enriched in the SECTM1 high-expression group, and TGF-β1 was positively correlated with SECTM1. The mRNA encoding SECTM1 was positively correlated with the mRNA expression of TGF-β1. Western blot analysis showed that the expression level of the TGF-β1/Smad signaling pathway was significantly down-regulated. These results indicated that SECTM1 regulated the proliferation, invasion and migration of GBM cells through TGF-β1/Smad signaling pathway.

Gliomas are associated with high morbidity and mortality. Abnormal regulation of the TGF-β/Smad pathway plays a crucial role in the pathogenesis of many cancers, including glioma^33^-^36^. Studies have confirmed that the TGF-β signaling pathway is activated in high-grade gliomas, promoting glioma cell growth and invasion^15^, exacerbating malignant behaviors of gliomas, such as stem-like properties^37^, vascularization^38^, and therapeutic resistance^39^. It is associated with survival and poor prognosis^40^, ^41^. TGF-β1, a crucial upstream trigger of the TGF-β/Smad pathway, was significantly up-regulated in both low-grade and high-grade gliomas compared to normal brain tissue^42^. Meanwhile, the TGF-β/Smad signaling pathway is a classical signaling pathway of EMT, and regulation of EMT-like processes can significantly increase the aggressiveness of GBM cells, leading to conventional treatment tolerance and poor prognosis^43^, ^44^. In addition, our analysis found that the TGF-β signaling pathway was enriched when SECTM1 was highly expressed, which proved that SECTM1 could positively regulate the expression of TGF-β1 and TGF-β receptor 2 (TGFβR2). These results suggest that SECTM1 may promote GBM invasion and EMT-like processes through the TGF-β1/Smad signaling pathway, leading to poor prognosis. Therefore, SECTM1 may be a potential target for GBM therapy.

In conclusion, our study found that SECTM1 is highly expressed in gliomas and is associated with survival and poor prognosis of GBM. After SECTM1 knockdown, the expression of TGF-β1 and TGFβR2 in GBM cell lines was down-regulated, and the TGF-β1/Smad signaling pathway was inhibited, thus inhibiting the proliferation, migration, invasion and EMT-like process of GBM. Interference with SECTM1 expression may prevent relapse or chemotherapy resistance in GBM, so SECTM1 may be a promising target for GBM treatment.

## Supplementary Material

Supplementary materials and methods, figures.

## Figures and Tables

**Table 1:**
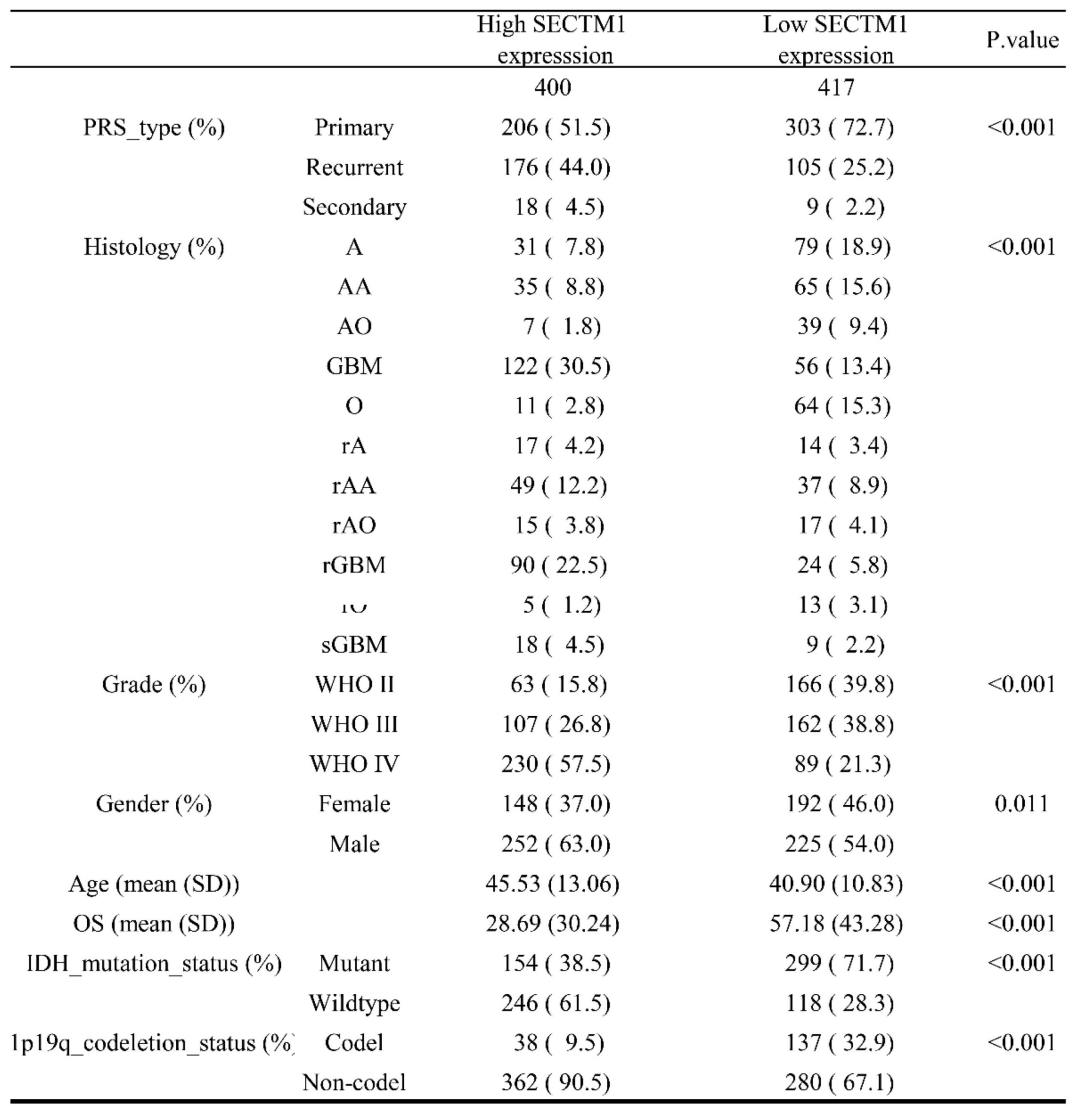
Correlation between glioma patients' different clinicopathological features and expression of SECTM1 in CGGA.
