# Supplementary material for: SECTM1 promotes the development of glioblastoma and mesenchymal transition by regulating the TGFβ1/Smad signaling pathway
Source: Int J Biol Sci. 2024 Jan 1;20(1):78–93. doi: 10.7150/ijbs.84591 (PMC10750278; doi:10.7150/ijbs.84591)
Supplement: Supplementary file 1 — Supplementary materials and methods, figures. [file ijbsv20p0078s1.pdf]

# SECTM1 promotes the development of glioblastoma and mesenchymal transition by regulating the TGFβ1/Smad signaling pathway

## Supplementary Materials and Methods

### Immunohistochemical (IHC) staining experiment

The expression levels of SECTM1 in human normal brain tissue and different grades of glioma were verified by immunohistochemical staining. The normal brain tissue paraffin sections and the tissue sections of Grade II to Grade IV glioma were obtained from the Department of Neurosurgery, Yijishan Hospital of Wannan Medical College. Paraffin tissue sections were dewaxed in xylene and rehydrated in graded alcohol, followed by routine IHC staining. 0.1 M citrate buffer (pH 6.0) was added to the slices and boiled for 15 min for antigen retrieval, then 3% $H_2O_2$ was dropped onto the tissues and incubated for 10 min in the darkroom to quench the endogenous peroxidase activity, non-specific binding was blocked by PBS with 5% BSA for 30 min at room temperature. Incubated with primary antibodies SECTM1 Monoclonal antibody (1:2000, 60281-1-Ig, Proteintech, Wuhan, China) or KI67 Polyclonal antibody (1:8000, 27309-1-AP, Proteintech, Wuhan, China) overnight at 4°C. Secondary antibodies were stained using immunohistochemical kits (Kgos60, Keygen Biotech, Nanjing, China). Nuclei were counterstained with hematoxylin solution and then cleared in alcohol and xylene. Observed and photographed under an upright microscope (Carl Zeiss Axio Scope Al, Germany).

### Quantitative real-time PCR (QPCR)

SECTM1 knockdown efficiency in cell lines U87 MG and U251 MG was verified by QPCR. The total RNA extraction from cell lines (U87 MG, U251 MG, LN229, KNS81, U87 MG-NC, U87 MG-sh1, U87 MG-sh2, U251 MG-NC, U251 MG-sh1, U251 MG-sh2) was performed when the cell density is about 90% using TRIzol reagent (10296-010, Invitrogen, Carlsbad, CA, USA), RNA extraction from tissues was performed according to the RNA extraction kit (DP431, Tiangen Biotech, Beijing, China). RNA

was reverse transcribed into cDNA by a cDNA synthesis kit (KR116, Tiangen Biotech, Beijing, China). Then, the QPCR was performed with the SuperReal PreMix Plus (SYBR Green) kit (FP205, Tiangen Biotech, Beijing, China). The primer sequences are as follows:

GAPDH forward: 5′- CAACTACATGGTTTACATGTTC -3′, GAPDH reverse: 5′- GCCAGTGGACTCCACGAC -3′, SECTM1 forward: 5′- CTTGGGACCCTCCTGTTTTT -3′, SECTM1 reverse: 5′- GCAGCTTGATGTTGACATGG -3′. TGF β 1 forward: 5′- GGCCTTTCCTGCTTCTCAT -3′, TGF β 1 reverse: 5′- GTCCTTGCGGAAGTCAATGT -3′,

**Western blot (WB)**

The expression of SECTM1 in human normal brain tissue and different grades of glioma and SECTM1 knockdown efficiency in cell lines U87 MG and U251 MG were determined by WB. Total protein extraction from human tissues and lentivirus-transfected U87 MG, and U251 MG cells using RIPA lysis buffer and separated by 10% SDS-PAGE, then transferred to polyvinylidene difluoride (PVDF) membrane. The membrane was blocked with 5% skim milk at room temperature for 2 hours and then incubated with primary antibodies with 1:2000 dilution of SECTM1 monoclonal antibody (60281-1-Ig, Proteintech, Wuhan, China), TGF Beta 1 Polyclonal antibody (1:2000, 21898-1-AP, Proteintech, Wuhan, China), TGFBR2 Monoclonal antibody (1:5000, 66636-1-Ig, Proteintech, Wuhan, China), anti-Smad2 (phospho S467) (1:1000, ab280888, Abcam), anti-Smad3 (phospho S423 + S425) (1:2000, ab52903, Abcam), E-cadherin Polyclonal antibody (1:10000, 20874-1-AP, Proteintech, Wuhan, China), Vimentin Monoclonal antibody (1:50000, 60330-1-Ig, Proteintech, Wuhan, China) or GAPDH (1:2500, ab9485, Abcam) overnight at 4°C. The membrane was incubated with secondary antibody HRP-conjugated Goat Anti-Mouse IgG(H+L) (1:5000, SA00001-1, Proteintech, Wuhan, China) or HRP conjugated goat anti-rabbit IgG (1:5000, ab181662, Abcam) at room temperature for 1 h. Finally, chemiluminescence signals of the target proteins were developed using an ECL detection kit (KeyGen Biotch) and

visualized and analyzed using ImageQuant 800 (GE, USA).

**Immunofluorescence staining (IF)**

Immunofluorescence staining verified the expression level of SECTM1 in normal human brain tissue and gliomas of different grades, as well as in normal human cells (HA) and glioma cells (U87 MG, U251 MG, LN229, KNS81). The normal brain tissue paraffin sections and the tissue sections of Grade II to Grade IV glioma were obtained from the Department of Neurosurgery, Yijishan Hospital of Wannan Medical College. Cell lines were seeded into a 6-well culture plate at a density of $5\times10^5$ cells per well and cultured in 5% $CO_2$ at 37°C for 24 h. then cells and tissue sections were fixed with 4% paraformaldehyde for 15 min and washed three times in PBS. Incubated with primary antibodies with 1:2000 dilution of SECTM1 monoclonal antibody (60281-1-Ig, Proteintech, Wuhan, China) overnight at 4°C and washed three times in PBS. Secondary antibodies were stained using goat anti-mouse IgG H&L (Cy5 ®) (ab6563, Abcam) and washed three times in PBS. The slides and cells were finally sealed with an anti-fluorescence quenching sealing solution containing DAPI (P0131, Beyotime Technology) and observed under a fluorescence microscope (Carl Zeiss Axio Scope Al, Germany).

**Supplementary Figures**

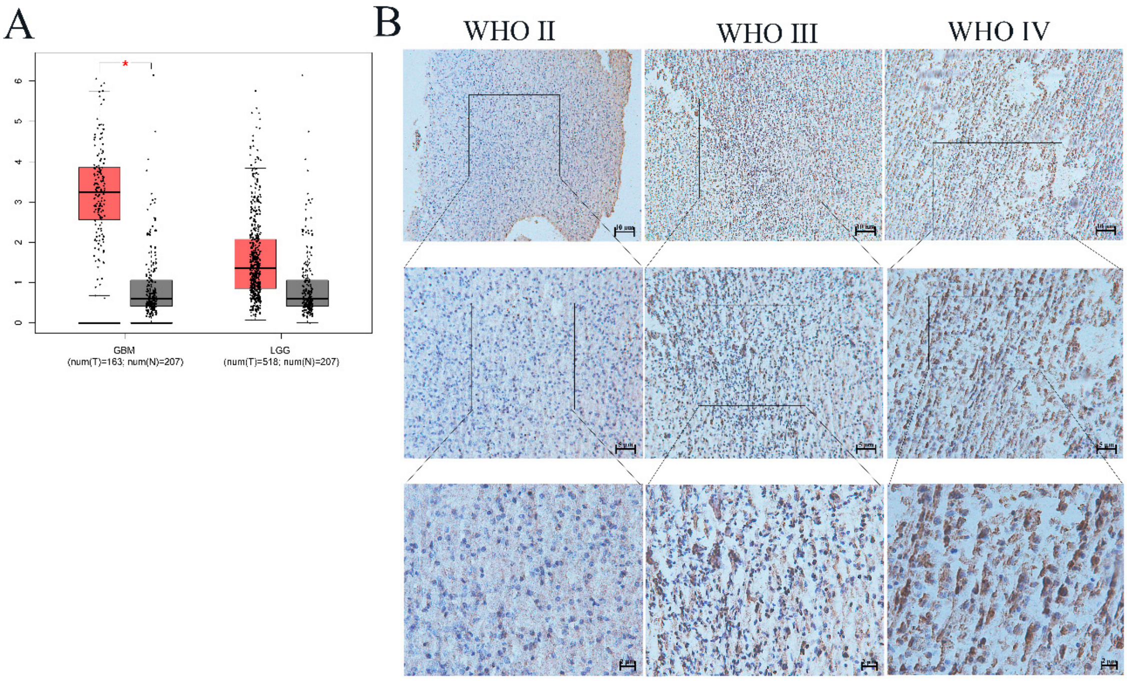


Figure S1. (A) Differences in SECTM1 expression between glioma and normal groups in the GTEx dataset. (B) Immunohistochemical analysis of SECTM1 expression in different grades of clinical glioma specimens. (scale bars were 10 μm, 5 μm, 2 μm, respectively).

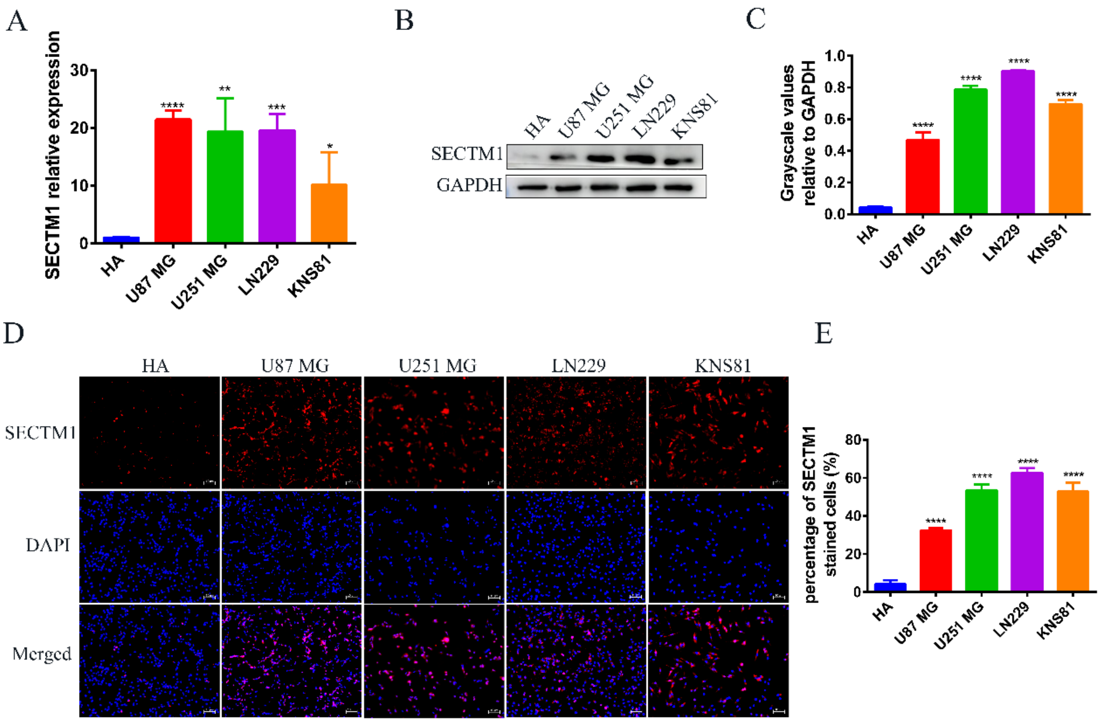


Figure S2. The expression of SECTM1 in the GBM cell line was detected. A-C was RT-qPCR (A) or Western blot (B-C) to detect the relative expression difference of SECTM1 in human astrocytes (HA) and human GBM cell lines (U87 MG, U251 MG, LN229, KNS81). D-E was used to detect the relative expression difference of SECTM1 in human astrocytes (HA) and human GBM cell lines (U87 MG, U251 MG, LN229, KNS81) by immunofluorescence staining (scale, 10μm). All data were analyzed by T-test for P-values as the mean ±SEM of three independent experiments. *P < 0.05; ** P <0.01; *** P < 0.001; **** P < 0.0001.

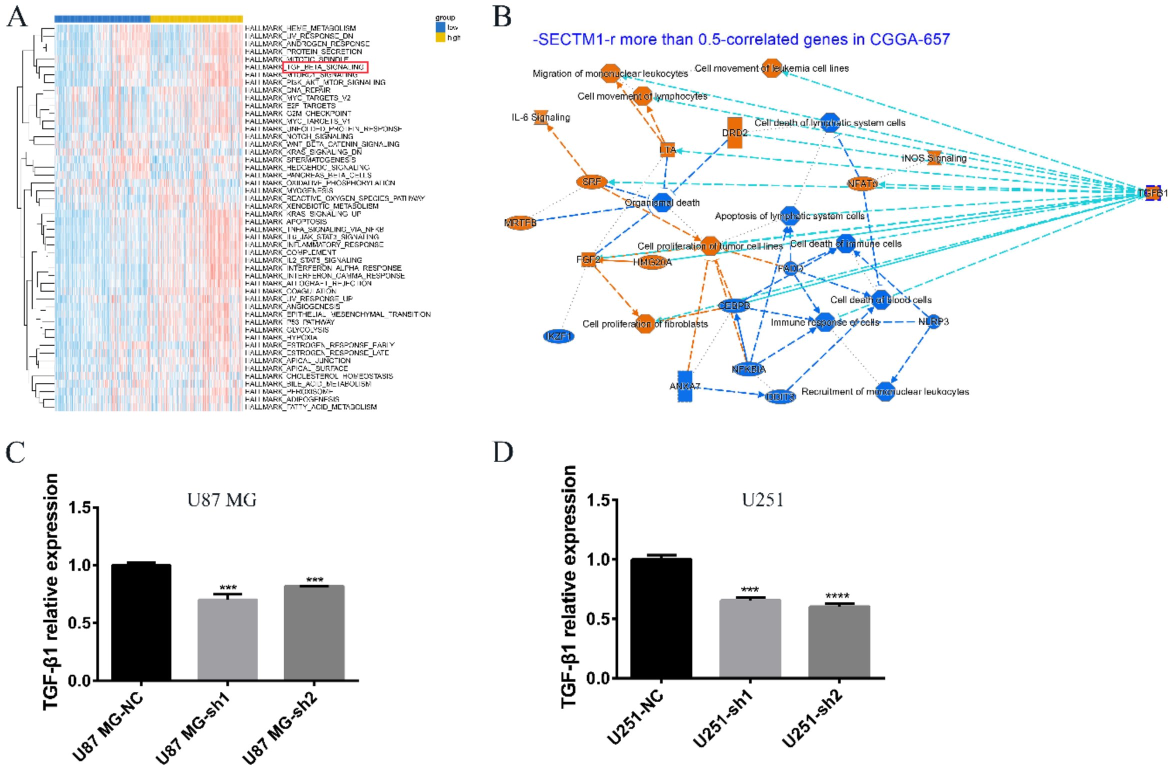


Figure S3. Signal pathway and functional enrichment analysis of SECTM1. (A) GSEA enrichment analysis of signal pathways that SECTM1 may be involved in regulation. (B) SECTM1-related genes were analyzed in CGGA. (C, D) qPCR was used to detect the expression of TGFβ1 gene in U87 MG cells (C) and U251 MG cells (D) after SECTM1 knockdown, respectively.

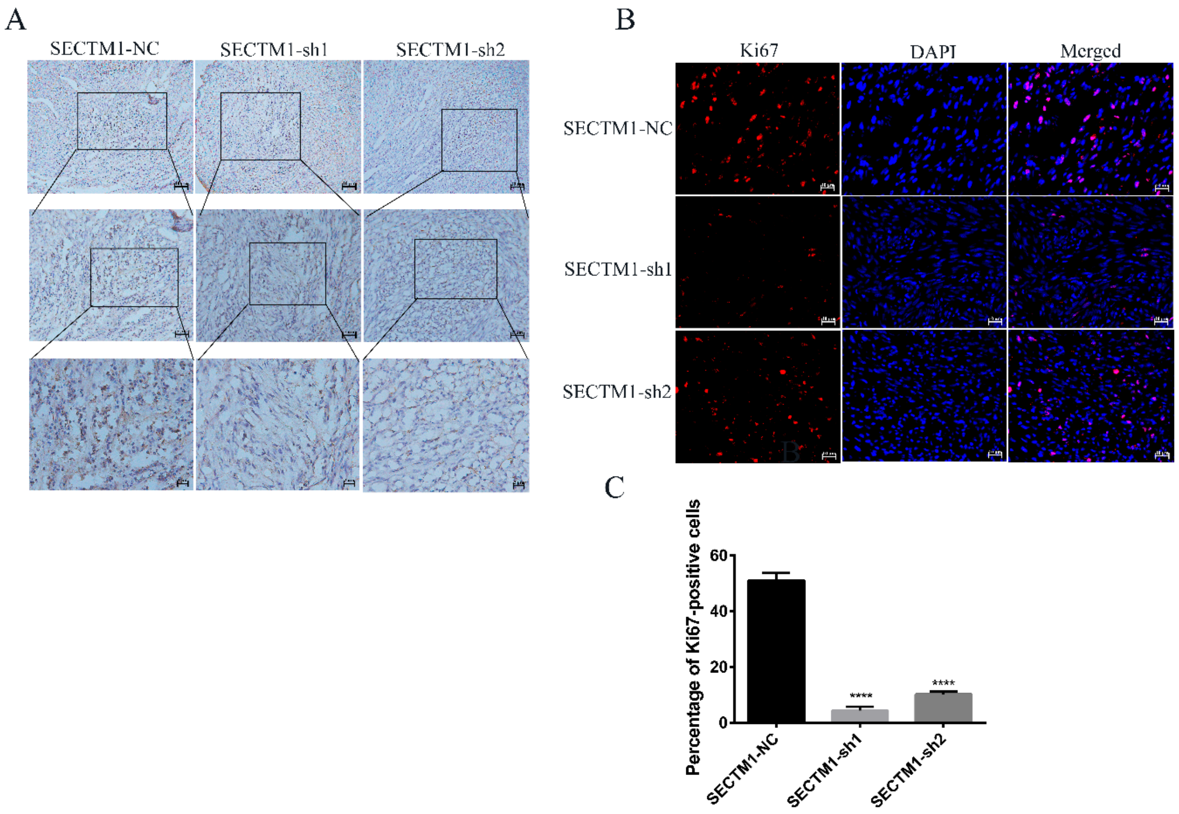


Figure S4. Effect of SECTM1 knockdown in U87 MG on tumorigenicity in mice. (A) The expression of SECTM1 in mouse tumor tissue was detected by immunohistochemistry. (B, C) The expression of Ki67 in mouse tumor tissues was detected by immunofluorescence, where C was quantitative immunofluorescence analysis.

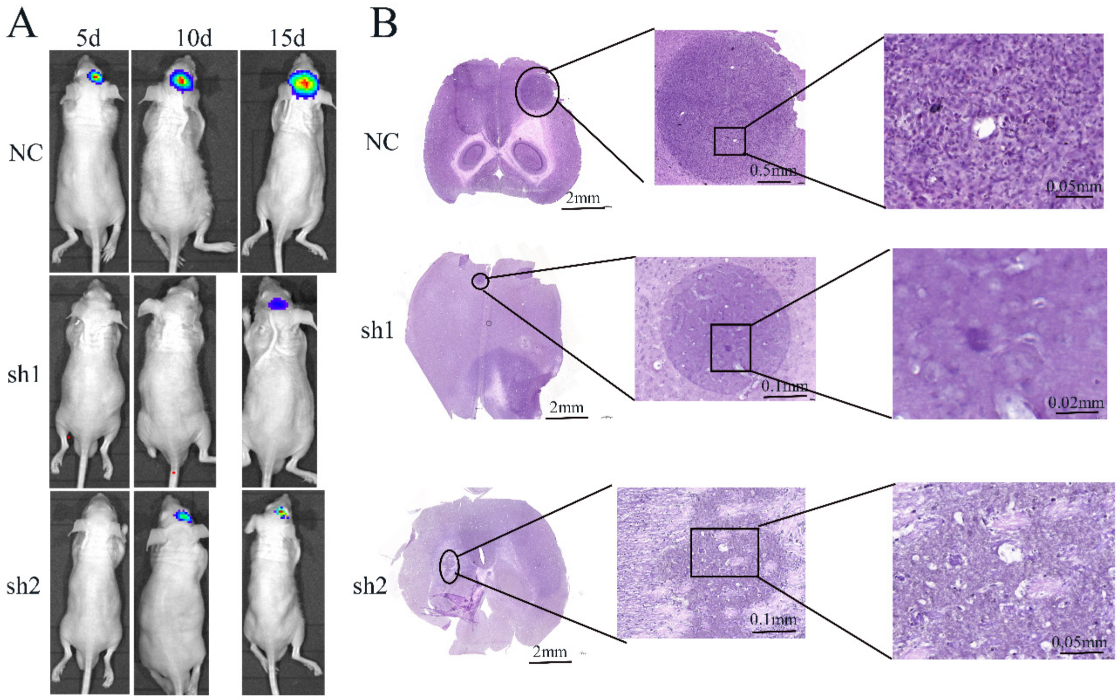


Figure S5. Effects of knockdown of SETM1 in U87 MG on in situ tumorigenesis in mice. (A) In vivo bioluminescence imaging of mice detected every 5 days after 7 days of in situ tumorigenesis in different cells (U87 LUC-NC, U87 LUC-sh1, U87 LUC-sh2, n=4). (B) HE-stained images of frozen sections of brain tissue from different groups of mice.

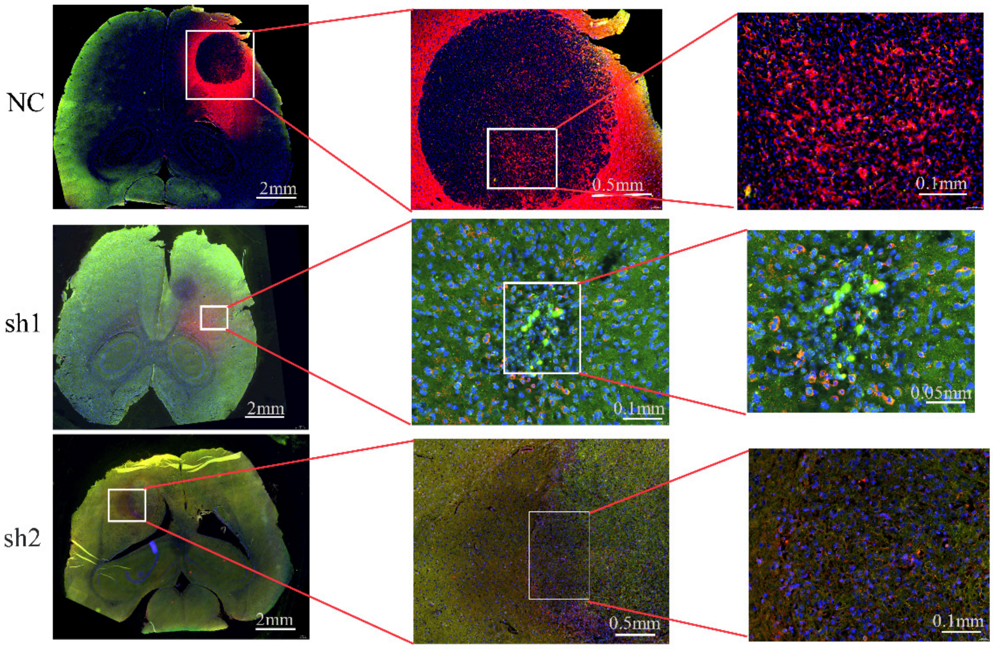


Figure S6. EMT markers immunofluorescence staining images of in situ tumor-bearing mice brain tissue frozen sections from different groups. E-cadherin: green; Vimentin: red; DAPI: blue.

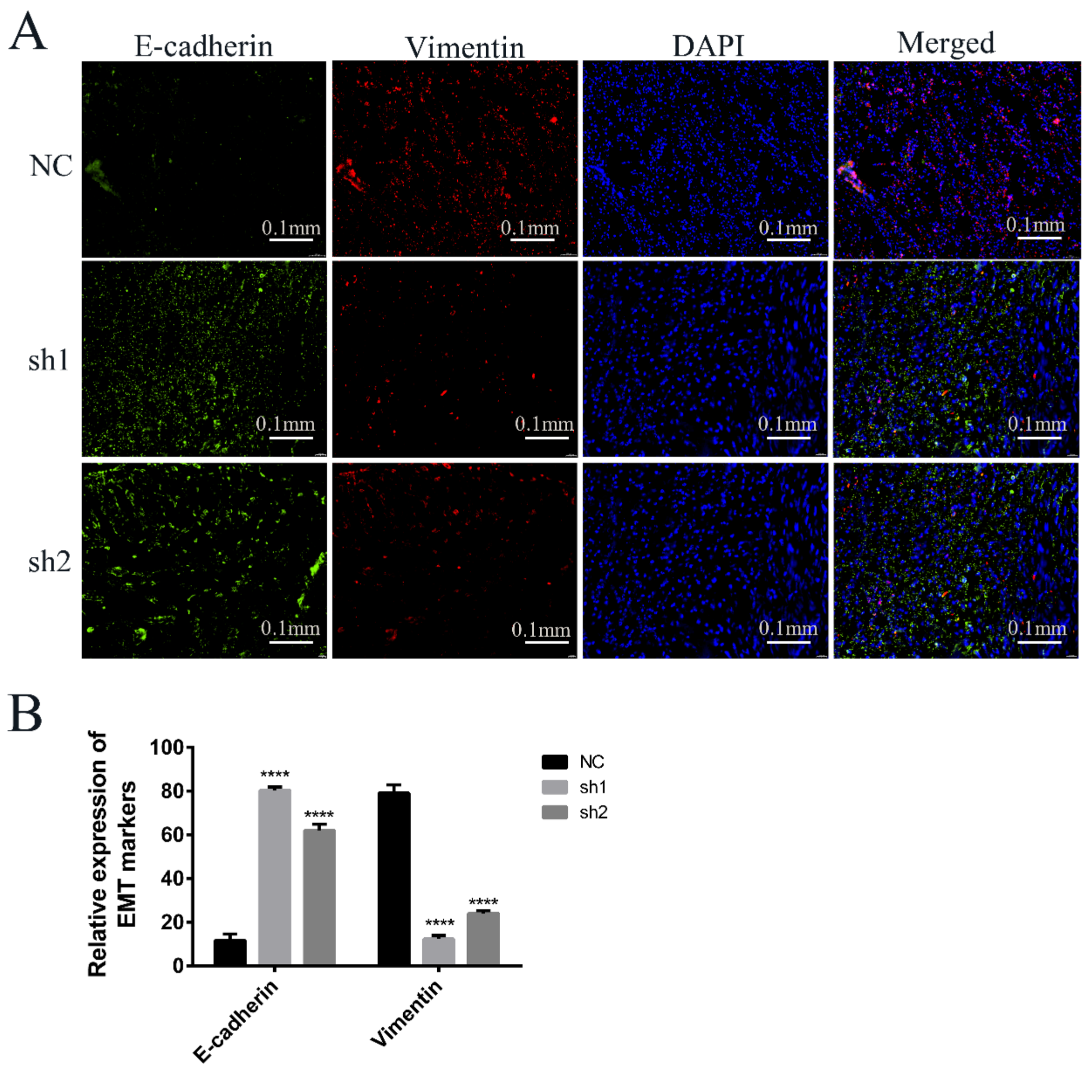


Figure S7. (A) EMT markers immunofluorescence staining images of subcutaneous tumor-bearing mice brain tissue frozen sections from different groups. (B) Quantitative analysis of EMT markers immunofluorescence staining results. The data represent the mean ±SEM of the T-test analysis through three independent experiments, **** $P < 0.0001$.

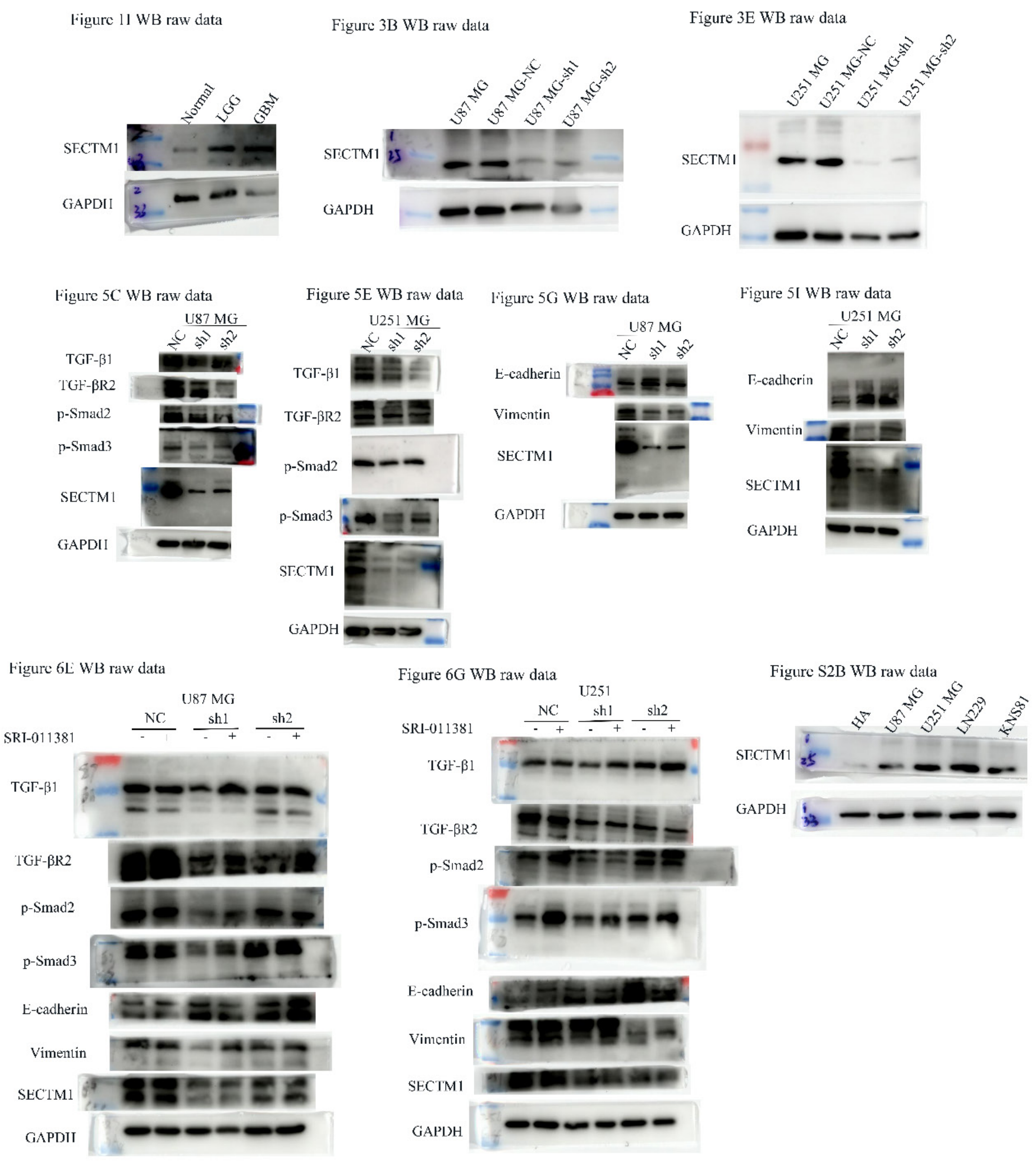


Figure S8. The raw WB data involved in this study.
